# Population-based multistate modelling of peritraumatic opioid use among trauma patients from the Norwegian Trauma Registry^☆^

**DOI:** 10.1016/j.bja.2025.05.008

**Published:** 2025-07-04

**Authors:** Sophia Engel, Leiv Arne Rosseland, Martijn Smits, Svetlana O. Skurtveit, Jon Michael Gran, Trond Nordseth, Olav Røise, Henrik B. Jacobsen

**Affiliations:** 1 Department of Research and Development, Division of Emergencies and Critical Care, Oslo University Hospital, Oslo, Norway; 2 The Mind Body Lab, Department of Psychology, University of Oslo, Oslo, Norway; 3 Institute of Clinical Medicine, Faculty of Medicine, University of Oslo, Oslo, Norway; 4 Department of Chronic Diseases, Division of Mental and Physical Health, The Norwegian Institute of Public Health, Oslo, Norway; 5 Norwegian Centre for Addiction Research (SERAF), Institute of Clinical Medicine, University of Oslo, Oslo, Norway; 6 Oslo Centre for Biostatistics and Epidemiology, Department of Biostatistics, Institute of Basic Medical Sciences, University of Oslo, Oslo, Norway; 7 Department of Anesthesia and Intensive Care Medicine, St. Olavs Hospital, Trondheim, Norway; 8 Department of Circulation and Medical Imaging, Norwegian University of Science and Technology, Trondheim, Norway; 9 Norwegian Trauma Registry, Division of Orthopedic Surgery, Oslo University Hospital, Oslo, Norway; 10 Department of Pain Management and Research, Oslo University Hospital, Oslo, Norway

**Keywords:** long-term opioid use, multistate model, persistent opioid use, short-term opioid use, traumatic injury

## Abstract

**Background:**

Previous studies document high rates of long-term opioid use (LTU) among trauma patients, without accounting for opioid prescription doses, frequency, and medical indications. Using detailed LTU criteria, this study aimed to construct a multistate model of peritraumatic opioid use and elucidate reasons for posttraumatic LTU.

**Methods:**

This population-based cohort study links data from the 2015–2018 cohorts in the Norwegian Trauma Registry (*n*=24 224) with outpatient medication prescription records from the Norwegian Prescription Database for 1 yr before and 2 yr after injury. LTU was assigned upon receiving ≥900 morphine milligram equivalents within 90 days and another opioid prescription 91–180 days after any initial prescription; it ended after 180 days without opioid prescriptions, emigration, or death. Prescriptions to patients with LTU in the year after injury were screened for chronic pain and palliative care reimbursement codes, and for opioid maintenance treatment preparations.

**Results:**

Posttraumatic opioid prescriptions primarily increased as a result of short-term opioid use (STU), peaking at 38.2% and returning to pre-injury levels (5.4%) within 6 months. Increases in LTU were smaller (4.3% to 6.7%). Among patients with LTU in the year after injury (*n*=1946), 47.4% exhibited LTU and 18.3% STU in the 6 months pre-injury. Moreover, 36.0% were reimbursed for chronic pain medications, 5.9% for palliative care, and 13.1% received opioid maintenance treatment.

**Conclusions:**

In our cohort, most patients with posttraumatic long-term opioid use had used opioids before injury, underscoring the need to address such use to minimise posttraumatic long-term opioid use. Few patients with long-term opioid use received palliative care or opioid maintenance treatment, indicating subacute and chronic pain as primary indications. As long-term opioid use for pain is highly controversial, findings emphasise a need for effective nonopioid alternatives.

**Editor’s key points**

• Many trauma patients are discharged with moderate to severe pain and continue to be prescribed opioids despite stricter opioid prescribing guidelines.
• Based on national data from Norwegian trauma and medication registries, this study provide an in depth assessment of peritraumatic short-term and long-term opioid use.
• Their analysis shows that trauma contributes to new cases of long-term opioid use, and that most individuals with posttraumatic long-term opioid use have used weak or strong opioids before injury.
• They also show that long-term opioid use can be sustained by opioids of any potency, and that pain appears the major indication for long-term opioid use which is highly controversial.
• The present study thus highlights that opioid stewardship in trauma care should take into account patients' pre-traumatic opioid use, encompass all opioids regardless of their potency, and at all times be guided by a clear medical indication for their prescription.

Many trauma patients experience moderate to severe pain at hospital discharge.^1^ Despite a growing awareness of opioid hazards and increasingly restrictive prescription guidelines, opioids remain an established treatment for managing acute pain.^2^^,^^3^ Opioid prescriptions at hospital discharge, however, considerably increase trauma patients’ risk to develop long-term opioid use (LTU),^4^ affecting up to 27% and 21% of those with^4^, ^5^, ^6^, ^7^, ^8^, ^9^, ^10^, ^11^, ^12^ and without^13^, ^14^, ^15^, ^16^, ^17^, ^18^ pre-traumatic opioid exposure, respectively. Trauma patients with LTU have an increased risk for opioid abuse,^3^ chronic pain,^19^ psychological distress,^19^ further traumatic injuries,^20^ and death.^11^

Previous studies on peritraumatic opioid use generally focus on specific trauma subpopulations (e.g. orthopaedic, military),^8^^,^^10^^,^^14^^,^^16^, ^17^, ^18^ or are restricted to individual trauma centers^4^^,^^5^^,^^9^^,^^18^ or specific injury types.^5^^,^^6^^,^^12^^,^^13^^,^^15^^,^^17^^,^^18^ Consequently, the generalisability of their results to the general trauma population may be limited. Additionally, many either do not specify the types of opioids studied,^4^^,^^7^, ^8^, ^9^^,^^15^^,^^16^ or focus solely on analgesic opioids,^6^^,^^11^, ^12^, ^13^^,^^17^^,^^18^ excluding those commonly utilised in opioid maintenance treatment (OMT). However, considering the overrepresentation of substance use disorder among trauma patients,^11^ excluding OMT opioids likely leads to an underestimation of the true LTU prevalence in this patient population. Furthermore, previous LTU definitions usually focus solely on the timing of opioid prescription,^4^, ^5^, ^6^, ^7^, ^8^, ^9^, ^10^, ^11^, ^12^, ^13^, ^14^, ^15^, ^16^, ^17^, ^18^ such as a single prescription occurring 90–180 days posttrauma, without considering other relevant aspects such as dose and frequency. As a single opioid prescription may only cover a short period and not necessarily indicate ongoing use, relying solely on timing may misclassify short-term opioid use (STU) as LTU, potentially overestimating the prevalence of LTU. Moreover, by solely differentiating between previously opioid-exposed and opioid-naïve patients, previous definitions may not account for pre-traumatic opioid use patterns. Accordingly, it is uncertain whether reported prevalences reflect new, potentially trauma-related, LTU, or a continuation of pre-injury usage. Finally, long-term opioid treatment for chronic non-malignant pain is controversial.^3^ Concerns include, among others, potential for dosage escalation, side-effects, misuse, addiction, and questionable long-term efficacy.^3^ However, long-term opioid treatment might be indicated and effective in palliative care^21^ or OMT.^22^ The medical indication for long-term opioid treatment is thus essential to derive valid conclusions about its appropriateness. Taken together, a clear overview of the prevalence and evolution of peritraumatic opioid use, and medical indications for posttraumatic LTU is lacking. Norway maintains high-quality population registries, including dedicated ones for trauma care and outpatient medication prescriptions, enabling comprehensive analyses to address these knowledge gaps.

We applied detailed criteria for STU and LTU that, in addition to timing, also account for prescription dose, frequency, and relevant competing events, such as death. Using recent population-based trauma and outpatient medication prescription data, the primary aim of this study was to investigate patterns of peritraumatic opioid use (no opioid use [NU], STU, LTU) from 6 months before to 18 months after injury using a multistate modelling approach. Secondary goals included characterising STU and LTU by types, frequency, and dose of opioid prescriptions, comparing opioid prescription aspects among patients with varying injury severities, and exploring possible medical indications for LTU in the first posttraumatic year. Given the applied LTU criteria’s level of detail, it was hypothesised that the prevalence of LTU, both before and after trauma, would be lower than previously reported.^4^, ^5^, ^6^, ^7^, ^8^, ^9^, ^10^, ^11^, ^12^, ^13^, ^14^, ^15^, ^16^, ^17^, ^18^

## Methods

This population-based cohort study includes injured patients registered in the Norwegian Trauma Registry (NTR) between January 01, 2015, and December 31, 2018. Reporting follows the Strengthening the Reporting of Observational Studies in Epidemiology (STROBE) guidelines.^23^ The study protocol was registered at Open Science Framework (OSF) Registries.^24^

As a part of the Injury Prevention and Outcomes following Trauma (IPOT) project, this study has been approved by the Regional Committee for Medical and Health Research Ethics (#2018/2010) and the data protection officer at Oslo University Hospital (#18/25315). The Norwegian Data Protection Authority has authorised the NTR to collect data from eligible patients without explicit consent. Hospitals inform affected patients about the possibility to withdraw their consent.

### Study setting and patients

**Fig 1:**
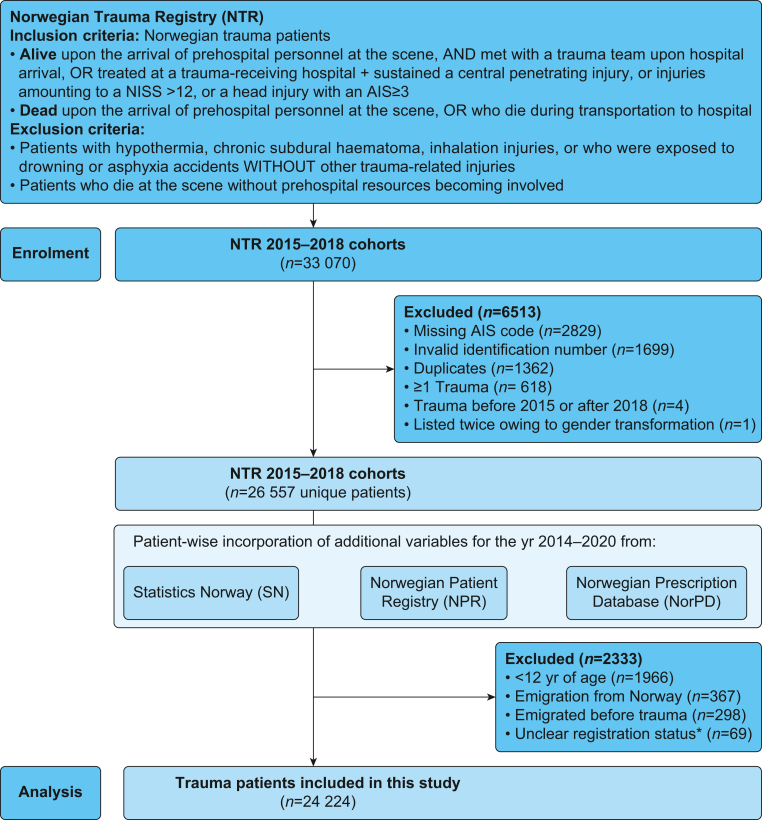
Database and patient flow of the present study. ∗Including unregistered patients and those with erroneous registry entries, or registered as born, but not living in Norway. AIS, Abbreviated Injury Scale; NISS, New Injury Severity Score.

In Norway, trauma care is provided by four regional trauma centres, offering level I or II trauma services according to American College of Surgeons Committee on Trauma standards, alongside 34 hospitals with trauma functions.^25^ All Norwegian trauma-receiving hospitals are obliged to register all eligible patients in the NTR. Between 2015 and 2018, NTR hospital coverage exceeded 85% and individual coverage improved substantially (missing cases: 2015 ≈ 40%, 2016 ≈ 20%, 2017/2018 <10%).^25^ NTR data are collected from patients’ medical journals by certified registrars and cover demographics, pre-traumatic comorbidities, and pre- and in-hospital trauma variables.^26^ The gender variable concerns patients’ legal gender at trauma. Norwegian legislation operates a binary gender definition (‘woman’ or ‘man’). NTR inclusion and exclusion criteria are provided in Figure 1.

Using the personalised identification number assigned to Norwegian residents upon birth or immigration, NTR data were linked to three Norwegian health registries for the period from January 01, 2014, to December 31, 2020.^26^ Altogether, this provides a shared follow-up duration of 1 yr before to 2 yr after injury for all included patients. Data accessed from the resulting database (Fig. 1) included: (1) data on outpatient prescriptions of prescription medication from the Norwegian Prescription Database (NorPD)^27^; (2) information on patient migration from Statistics Norway^28^; and (3) surgical codes registered in the Norwegian Patient Registry.^29^

Because most opioid types are recommended for individuals aged ≥12 yr, patients below that age were omitted from the analyses. Trauma patients registered as emigrated or with an ambiguous pre-trauma residential status were excluded, as medication prescription data are likely to be incomplete in these instances (Fig. 1).

### Opioids

All opioids prescribed for analgesia (Anatomical Therapeutic Chemical [ATC]^30^ group N02A) and OMT (ATC-group N07BC) dispensed in Norway between 2014 and 2020 were considered. As opioids differ in potency and strength, relevant prescriptions were converted to equianalgesic doses of morphine expressed in morphine milligram equivalents (MMEs) to enable valid comparisons. Supplementary Table S1 provides an overview of included opioid types and applied conversion factors. In Norway, codeine is typically prescribed in combination with paracetamol for pain (ATC-code N02AJ06). When mentioning codeine, we refer to this combination.

### Criteria for no, short-, and long-term opioid use

Standardised definitions for STU and LTU are still lacking. This study applies a modified version of criteria from a recent study using NorPD data.^31^ Outlined below are the criteria and the reasoning behind their selection.

#### Long-term opioid use

Patients receiving ≥900 MME within 90 days (i.e. ≥10 MME per day), starting from any initial opioid prescription, and filling another opioid prescription 91–180 days after that initial prescription were classified as exhibiting LTU. The 90-day criterion was selected based on the definition of chronic pain^32^ and evidence indicating that opioid prescriptions exceeding 90 days substantially increase the risk of developing opioid use disorder.^3^ To ensure that patients had an opioid supply sufficient to last at least 90 days, we set a threshold of 10 MME per day on average (≥900 MME per 90 days), which constitutes a conservative dosage. To confirm that opioid usage lasted beyond 90 days, we required at least one additional opioid prescription between days 91 and 180 after the initial prescription. Periods of LTU ended when either 180 days without an opioid prescription elapsed, or when patients emigrated from Norway (resulting in loss to follow-up), or died.

#### Short-term opioid use

All opioid prescriptions not meeting LTU criteria were considered STU.

#### No opioid use

All individuals who did not receive an opioid prescription during any given study quarter were classified as NU.

**Fig 2:**
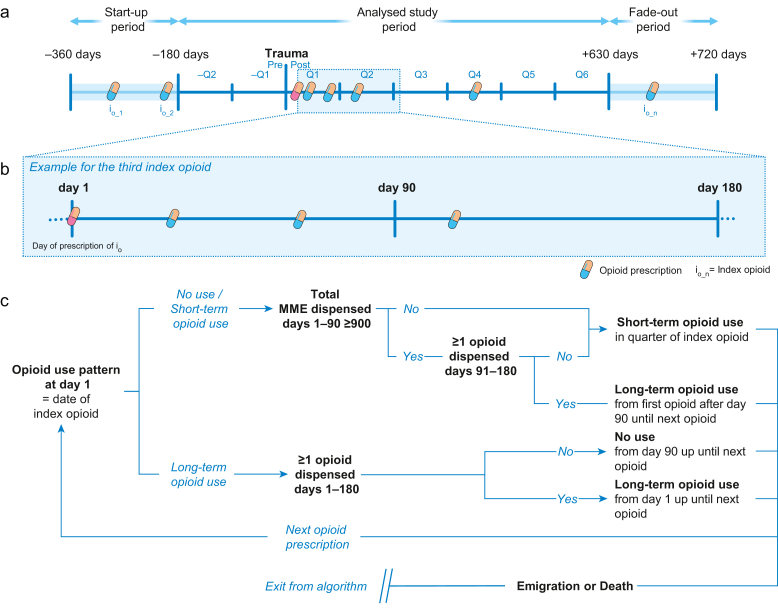
Criteria and algorithm applied to construct a multistate model of peritraumatic opioid use. (a) Visualisation of the algorithm which, in a patient-wise manner, screened all consecutive outpatient medication prescriptions registered at the Norwegian Prescription Database during the 360 days before and 720 days after trauma for relevant ATC codes of opioids. As the applied criteria require a minimum of 91 days to initiate long-term opioid use, the first and last 180 days of the study period were censored to prevent left truncation bias. (b) For every opioid prescription, the algorithm applied a set of criteria where the opioid prescription itself (= index opioid [i_o_]) marked day 0. Criteria related to the subsequent 180 days. (c) Visualisation of the criteria applied by the algorithm to every index opioid to define patients’ current opioid use pattern; no, short-, or long-term use. Once a respective patient's usage pattern was established, the algorithm applied the same criteria to the subsequent opioid prescription. This process was repeated until the last opioid prescription during the study period was reached. Emigration from Norway and death were registered as relevant events competing with opioid prescription and to monitor loss to follow-up. As chosen criteria allow individuals to transition between different patterns of opioid use over time, this can be considered a multistate modelling approach. Q, quarter (90 days); MME, morphine milligram equivalents.

Please see Figure 2 for a schematic overview of the algorithm developed to apply these criteria to the dataset.

### Medical indications for posttraumatic long-term opioid use

To investigate medical indications for LTU, prescription data of patients exhibiting LTU during the first posttraumatic year were screened for diagnostic codes signifying potential medical indications for long-term opioid treatment, including medication codes specific to the opioid types typically used in OMT (ATC-group N07BC) and national reimbursement codes that indicate the dispensing of medications for either palliative care (‘-90’) or chronic non-malignant pain (‘-71’).

### Statistical analyses

Statistical analyses were performed using R software version 4.2.3.^33^ The statistical code of the multistate model of peritraumatic opioid use and associated synthetic dataset are accessible at https://doi.org/10.17605/OSF.IO/CXKR3.

The overall proportion of missing values for included variables was 0.29%. As this study relies on population data, power calculations were dispensable. The multistate model of peritraumatic opioid use, constructed based on the rules described above, was summarised in an ‘alluvial plot’. Although opioid use patterns were assigned consecutively for each day of the study period, it was decided to summarise them per quarter to better capture general trends. To characterise STU and LTU, between-group differences in dispensed opioid types and opioid prescription frequency per quarter were summarised in a tabloid figure. Additionally, STU and LTU patients’ average daily MME supply per study quarter was visualised in scatterplots. Owing to questionable reliability, MME quantities for N07BC-opioids were omitted from the plot. We also created a line chart to summarise differences in the median MME provided to STU and LTU patients, comparing those with non-severe (Injury Severity Score [ISS] ≤15) *vs* severe injuries (ISS >15). For patients with LTU, we created an additional table displaying the mean and median duration of opioid use before and after trauma, again with comparisons drawn between patients with non-severe injuries and those with severe injuries. The prevalence of potential medical indications for LTU among patients exhibiting LTU during the first posttraumatic year was summarised in a second alluvial plot.

### Sensitivity analyses

Considering the NTR’s lower trauma patient coverage during 2015–2016 compared with 2017–2018, independent samples *t*-tests and χ^2^ tests were conducted to assess potential baseline differences between patients from these periods. Effect sizes were assessed using Cohen's d (*t*-tests) and Cramer's V (χ^2^ tests). To explore the impact of different MME criteria on LTU proportions, two additional alluvial plots were created: one with a criterion of ≥5 MME per day and another one with a criterion of ≥50 MME per day for the first 90 days of LTU. The ≥50 MME per day criterion also illustrates the proportions of patients at an elevated risk for opioid overdose.^34^ To examine whether weight-based opioid dosing for adolescents (age 12–17 yr) might have affected study results, we created an overview of all opioid types dispensed to adolescents, detailing administration methods, standard dosing according to Norwegian guidelines, and the frequency of prescription before and after trauma. To explore the impact of including adolescents on reported prevalences of peritraumatic opioid use (NU, STU, LTU), we compared the prevalence rates of the entire sample with those of the adult population (≥18 yr). Cramer's V was calculated to quantify the magnitude of the differences.

## Results

Please refer to Table 1 for a description of final study sample.

**Table 1:** Characteristics of the 24 224 trauma patients included in this study. 25^th^, 25^th^ percentile; 75^th^, 75^th^ percentile; AIS, Abbreviated Injury Scale; ASA, American Society of Anesthesiologists Physical Status Classification System; ICU, Intensive Care Unit; ISS, Injury Severity Score; *n*, Number of patients; NA, not applicable; NISS, New Injury Severity Score; sd, standard deviation. An AIS score ≥3 signifies that an injury is at least serious, but potentially severe, critical, or even unsurvivable. Where applicable, numbers of missing cases are indicated.

Variable	Mean (sd) or *n* (%)	Median (25th, 75th)	Missing, *n* (%)
**Demographics**			
Age (yr)	45.55 (22.14)	45 (25, 63, range: 12–102)	NA
Sex			
Female	7806 (32.2%)	NA	NA
Male	16 418 (67.8%)	NA	NA
**Comorbidity**			
ASA physical status 1	14 640 (60.4%)	NA	612 (2.5%)
ASA physical status 2	6427 (26.5%)	NA	
ASA ≥3	2545 (10.5%)	NA	
**Mechanism of injury**			
Traffic-related	11 352 (46.8%)	NA	653 (2.7%)
Fall	8493 (35.1%)	NA	
Other	3727 (15.4%)	NA	
**Injury characteristics**			
ISS			
Average	8.18 (8.79)	5 (1, 10)	NA
Minor (0–8)	14 544 (60.0%)	NA	NA
Moderate (9–15)	5819 (24.0%)	NA	NA
Severe (16–24)	2160 (8.9%)	NA	NA
Very severe (≥25)	1701 (7.0%)	NA	NA
NISS	10.57 (11.70)	6 (2, 14)	NA
Injured body part with AIS ≥3			
Head	3114 (12.9%)	NA	NA
Face	128 (0.5%)	NA	NA
Neck	106 (0.4%)	NA	NA
Thorax	3211 (13.3%)	NA	NA
Abdomen	821 (3.4%)	NA	NA
Spine	966 (3.4%)	NA	NA
Upper extremities	216 (0.9%)	NA	NA
Lower extremities	1656 (6.8%)	NA	NA
**Hospital stay**			
ICU admission	13 928 (57.5%)	NA	433 (1.8%)
Length of ICU stay (days)	1.62 (3.85)	1 (0, 2)	NA
Surgery within 2 weeks from injury	8502 (35.1%)	NA	NA
Length of hospital stay (days)	2.87 (6.65)	2 (0, 3)	276 (1.1%)

### Peritraumatic patterns of opioid use

**Fig 3:**
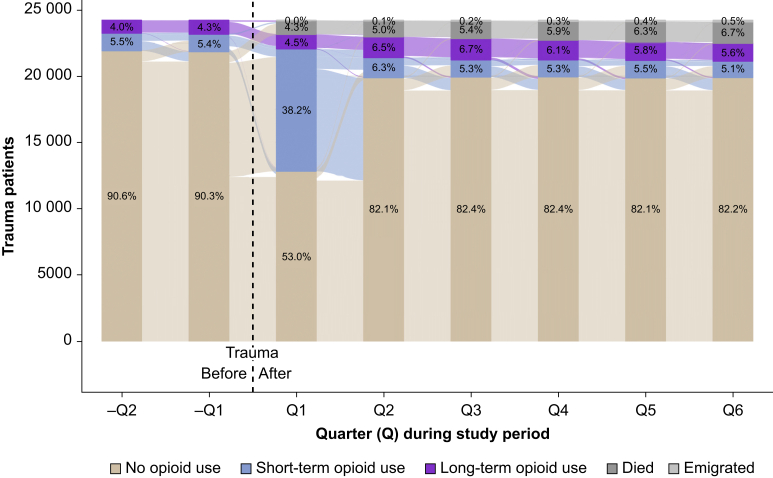
Multistate model of peritraumatic opioid use among 24 224 Norwegian trauma patients. This alluvial plot illustrates the progression of opioid use patterns (no, short-term, long-term) and competing events (death, emigration) from 6 months before to 18 months after trauma. The X-axis represents time in quarters (90-day periods), and the Y-axis shows the total number of patients. Each bar indicates the prevalence of different opioid use patterns and competing events per quarter. The flows between bars depict transitions of patients between states from one quarter to the next. Each flow is colour-coded based on the originating category and directed towards the destination category. The width of each flow corresponds to the number of patients making that specific transition; wider flows represent more common category changes.

Of the 24 224 patients included, 12.9% (*n*=3117) were dispensed opioids during the two quarters preceding trauma, 34.5% (*n*=1075) of whom met LTU criteria for at least one quarter. The multistate model of peritraumatic opioid use (Fig. 3) highlights that STU increased sharply during the initial quarter after injury, but then regressed to pre-injury levels within the two subsequent quarters. Posttraumatic increases of LTU were less pronounced and largest from the first to second quarter. Despite proportions of LTU decreasing thereafter, at the end, compared with beginning, of the study a net increase of 1.6% was observed. During each quarter, starting from the second posttraumatic one, >50% of patients being dispensed opioids classified as LTU. On average patients classified as LTU for 4.70 (sd 2.39) quarters. Few patients changed from LTU to STU or NU. A total of 2.3% (*n*=558) of the sample met LTU criteria during all analysed study quarters.

### Characteristics of short- and long-term opioid use

Among STU patients, codeine and tramadol accounted for ≥78.5% of prescriptions during all study quarters except for the first quarter after trauma, where a clear increase in oxycodone prescriptions was observed (22.3%) (Supplementary Fig. S2). Among LTU patients in contrast, oxycodone was the most dispensed opioid type throughout all posttraumatic study quarters, accounting for 18–26% of prescriptions. Following closely were codeine and tramadol, and opioid types primarily used in OMT, accounting for 17–18%, 19–22%, and 17–25% of prescriptions per quarter, respectively. On average, LTU patients filled opioid prescriptions 2.8 times more frequently than STU patients (Supplementary Fig. S2).

For any studied quarter, the median daily MME supply was considerably higher for LTU than for STU patients (Supplementary Fig. S3; Supplementary Table S4). STU patients’ median (interquartile range [IQR]) daily MME supply peaked during the first posttraumatic quarter (3.33 [IQR 1.67–7.48]), but then regressed to pre-injury levels within the subsequent one (Q3: 1.67 [IQR 0.83–3.33]). LTU patients displayed considerable subgroup differences. While those with chronic pain, and for whom a medical indication for LTU was not identifiable within our data were dispensed a median of 10–17 MME per day during any quarter, patients in palliative care received approximately twice that amount during posttraumatic quarters.

Among STU patients, those with severe injuries received higher opioid doses during the initial three posttraumatic quarters, and more varied opioid doses overall compared with those with non-severe injuries. In contrast, LTU patients with severe and non-severe injuries received similar opioid doses throughout the study period; however, the range of doses provided to patients with non-severe injuries was much larger (Supplementary Fig. S5). Additionally, the mean and median duration of LTU periods after trauma were considerably longer for patients with non-severe injuries than for those with severe injuries (Supplementary Table S6).

### Medical indications for posttraumatic long-term opioid use

**Fig 4:**
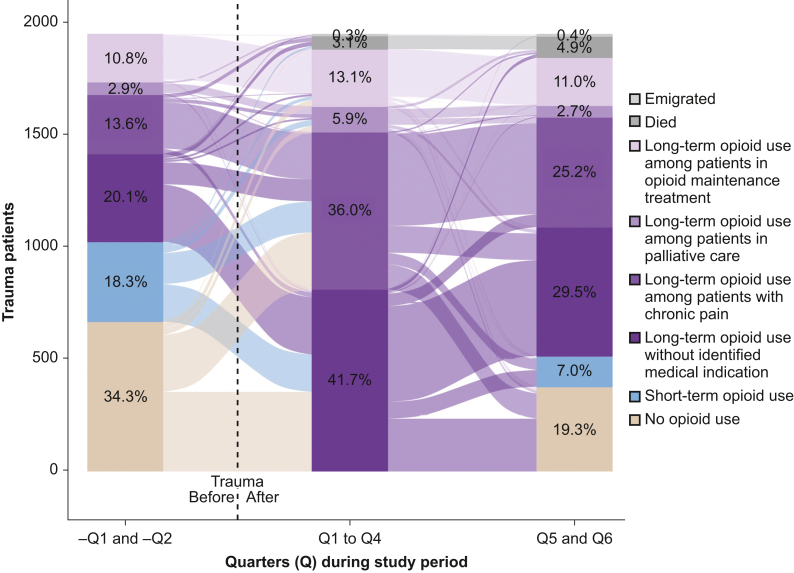
Medical indications for long-term opioid use (LTU). Based on data of all patients meeting LTU criteria during the first year after trauma (*n*=1946), this figure summarises the prevalence of medical indications for LTU, including chronic non-malignant pain, palliative care, and opioid maintenance treatment, for various quarters (90-day periods) of the study period. The X-axis represents time in quarters, and the Y-axis shows the total number of patients. Each bar indicates the prevalence of the different medical indications for LTU within a given time period. The states of no and short-term opioid use are included to show the proportions of patients with LTU who transition from or back to these states. Emigration and death are depicted in the figure as they are relevant events that compete with opioid prescription. The flows between bars depict transitions of patients between states from one quarter to the next. Each flow is colour-coded based on the originating category and directed towards the destination category. The width of each flow corresponds to the number of patients making that specific transition; wider flows represent more common category changes.

A medical indication for long-term opioid treatment was identified for 55% of patients with LTU in the first year after injury (Fig. 4). Among these, approximately 6% were receiving palliative care, 13% were in OMT, and 36% had chronic pain. The remaining 42% used opioids long-term for reasons other than those identifiable within our data.

### Sensitivity analyses

Sensitivity analyses comparing the 2015–2016 and 2017–2018 NTR cohorts revealed some statistically significant differences, but in all cases effect sizes indicated these to be inconsequential (Supplementary Table S7). Applying a criterion of ≥5 MME per day for the first 90 days of LTU increased LTU proportions by 1.0% for pre-traumatic and 0.7–1.4% for posttraumatic quarters (Supplementary Fig. S8). Applying a criterion of ≥50 MME per day for the first 90 days of LTU decreased LTU proportions by about 1.9–2.1% for pre-traumatic and 2.2–3.9% for posttraumatic quarters (Supplementary Fig. S9). All opioids dispensed to adolescents aged 12–17 yr were oral formulations. Weight-based opioid dosing potentially affected 5% of prescriptions to adolescents before trauma and 2.35% after trauma (Supplementary Table S10). Excluding adolescents increased STU and LTU proportions for each study quarter, with the most pronounced increases being 2.1% and 0.9%, respectively, during the first quarter after injury. For each study quarter, however, effect sizes indicated the differences between the two samples to be insignificant (Supplementary Table S11).

## Discussion

### Peritraumatic patterns of opioid use

Compared with other European countries, Norway has a higher than average incidence of opioid prescriptions per inhabitant and mortality after opioid overdose.^35^ Nevertheless, this study demonstrates that most Norwegian trauma patients are not dispensed opioids, neither before nor after injury. We find that posttraumatic increases in opioid prescriptions are mostly owing to STU and therefore temporary. LTU reached a maximum of 6.7% during the second posttraumatic quarter. Consistent with our hypothesis, this is below previously reported prevalences of LTU.^4^, ^5^, ^6^^,^^8^, ^9^, ^10^^,^^12^, ^13^, ^14^, ^15^, ^16^, ^17^, ^18^ Although one might argue that the inclusion of adolescents aged 12–17 yr in our study might have affected this result, our sensitivity analyses demonstrate that weight-based opioid dosing to adolescents was infrequent and that prevalence estimates changed insignificantly when excluding adolescents. Importantly, 53% of trauma patients with LTU during the first posttraumatic year had not used opioids during the 6 months before injury. Moreover, few patients with LTU transitioned back to STU or NU, keeping prevalence of LTU elevated until the end of the study period. Thus, although on a smaller scale than previous studies suggest, trauma contributed to novel LTU cases in our sample. In the absence of a standardised LTU definition, however, it is important to acknowledge that reported prevalence estimates are inherently reliant on the specific LTU criteria applied, making between-study comparisons challenging. This is also reflected in the results of our sensitivity analyses, where the proportion of patients meeting LTU criteria varied by a factor of 2–3 per quarter depending on the MME cut-off used.

### Characteristics of short- and long-term opioid use

The comprehensive criteria used in this study facilitate more detailed characterisations of peritraumatic opioid use than achieved previously. Specifically, STU was characterised by 1.6–2.2 opioid prescriptions per quarter, primarily involving codeine and tramadol and resulting in a median supply of 150 MME for each consecutive study quarter except the first posttraumatic one. This quantity corresponds to a 7-day supply of tramadol or a 12.5-day supply of codeine, when used at dosages recommended for treating moderate acute pain (tramadol 50 mg three times daily, codeine 30 mg four times daily). These characteristics, along with the substantial exchange observed between the STU and NU categories in the multistate model of peritraumatic opioid use, support that the applied STU definition accurately captures opioid use on an as-needed basis. The MME dispensed to STU patients increased sharply after trauma, with those experiencing severe injuries generally receiving higher amounts than those with non-severe injuries. For patients with non-severe injuries, MME dosages returned to pre-traumatic values within one quarter, whereas for those with severe injuries, the MME dispensed decreased linearly over three quarters. This finding aligns well with current opioid prescribing guidelines, which acknowledge that initial opioid treatment might be necessary to address moderate to severe acute pain, and it suggests adherence to reassessment and tapering protocols after the acute phase.^3^

In contrast, LTU was characterised by more frequent opioid redemptions, ranging 4.3–6.1 per quarter, with codeine, tramadol, and oxycodone accounting for, respectively, 16–18%, 19–22%, and 18–26% of prescriptions per quarter. Despite current opioid prescription guidelines focusing on dosing according to MME, the categorisation of opioids as ‘weak’ (e.g. codeine, tramadol) or ‘strong’ (e.g. oxycodone) persists in clinical practice and is often accompanied by a lower reluctance to prescribe ‘weak opioids’ because of their perceived lower harmfulness. Our results, however, highlight that opioids across varying potencies can facilitate and sustain LTU among trauma patients. Additionally, previous studies indicate that opioid potency is merely one among several biopsychosocial factors that collectively determine the risk of adverse events associated with opioid consumption.^36^ For physicians involved in trauma care, it is thus crucial to recognise that all opioids, whether ‘weak’ or ‘strong’, have the potential to cause misuse, abuse, addiction, and overdose, especially when prescribed for periods extending beyond 90 days.^3^ Furthermore, in our cohort 66% of patients exhibiting LTU during the first posttraumatic year had used opioids in the 6 months preceding their injury. This aligns with previous studies, identifying pre-traumatic opioid use as a key predictor of posttraumatic LTU,^4^^,^^7^^,^^10^, ^11^, ^12^ and suggests that evaluating pre-traumatic opioid use during initial hospitalisation is crucial and should inform ongoing pain management both in-hospital and after discharge. Interestingly, neither did the MME provided to LTU patients increase during the initial post-injury quarters, nor was there a difference in the amounts dispensed to those with severe and non-severe injuries. Instead, patients with non-severe injuries received a broader range of MME dosages and exhibited longer durations of LTU than those with severe injuries. This indicates that opioid dosing and prescription patterns for LTU patients may be influenced by factors other than injury severity, underlining the importance for physicians to understand the medical indications for long-term opioid treatment in their patients.

### Medical indications for posttraumatic long-term opioid use

Although LTU can be indicated in contexts such as OMT and palliative care,^21^^,^^22^ its use for treating subacute and chronic non-malignant pain is highly controversial, as associated risks and drawbacks outweigh the benefits for most patients.^3^ We were able to identify a medical indication for LTU for 55% of our cohort: 6% were receiving palliative care, 13% were receiving OMT, and 36% had chronic non-malignant pain. As pain is the major indication for opioid treatment, it is, however, reasonable to assume that a certain proportion of patients without an identifiable medical indication for LTU in our data were prescribed opioids long-term for subacute or chronic pain as well. Finding this group's median opioid dosages similar to that of chronic pain but not palliative care patients further supports this assumption. Our results thus suggest that subacute and chronic non-malignant pain is the primary reason for posttraumatic LTU among trauma patients. A recent Norwegian qualitative study showed that despite guidelines mandating re-evaluation of opioid treatment for acute pain after 4 weeks,^2^ several previously opioid-naïve trauma patients continued using opioids beyond 18 months after injury without reassessment.^37^ Given that trauma patients using opioids beyond 2 months after injury have been shown to experience increased pain, emotional distress, and disability,^19^^,^^38^^,^^39^ and considering the numerous risks associated with LTU,^3^ these findings are concerning. Against this background, we advocate for enhanced opioid stewardship for trauma patients. Citing the 2022 Centers for Disease Control and Prevention (CDC) guidelines,^3^ priority should be given to nonpharmacological and nonopioid strategies for acute pain management, with opioids considered only if these options prove inadequate and the benefits are anticipated to outweigh the risks for the individual patient. Moreover, given the multifaceted nature of posttraumatic pain, future research should concentrate on developing pain management strategies that provide trauma patients with effective alternatives to opioid treatment.

### Strengths and limitations

This study’s strengths include the NTR’s broad inclusion criteria and national coverage, suggesting high external validity. Moreover, utilising medication prescription data effectively mitigated self-reporting and recall bias. Additionally, assigning opioid use patterns in a time-to-event manner prevented immortal time bias. Lastly, considering the current lack of a uniform LTU definition, performed sensitivity analyses provide valuable insights about the dependence of presented results on specific aspects of applied LTU criteria.

Nevertheless, actual opioid use may have been underreported as NorPD covers neither prescription medication provided at inpatient facilities nor illicit drug use. Simultaneously, because of relying on medication prescription data, overreporting of actual opioid use cannot be excluded either. Yet, continued opioid prescription, as required to keep the LTU status in this study, strongly suggest continued use. The use of dispensing dates to define LTU may lead to the onset of LTU phases being influenced by trivial factors such as available pack size and prescribing habits. Identified medical indications for LTU might underrepresent patients in need of palliative care or chronic non-malignant pain treatment owing to delays or idiosyncrasies introduced by the application process for reimbursement codes. Moreover, it cannot be guaranteed that all patients dispensed OMT preparations truly receive OMT, as methadone is used for pain management in individual cases.

### Conclusions and implications

This study integrates population-based trauma data with high-quality outpatient medication prescription records and uses detailed criteria for STU and LTU, considering not just prescription timing but also dose, frequency, and relevant events competing with opioid use. Consequently, our study provides a more comprehensive understanding of the development and characteristics of peritraumatic opioid use than attained previously. Resonating with previous evidence, our results suggest that trauma contributes to new LTU cases and that most individuals with posttraumatic LTU have already used opioids before injury. This highlights the importance of assessing and managing pre-traumatic opioid use to effectively minimise posttraumatic LTU. Our data further indicate that opioid stewardship in trauma care should encompass all opioids, regardless of their potency. Finally, identifying pain as the main reason for LTU among trauma patients, alongside ongoing debates about potential risks and limited effectiveness of long-term opioid therapy for managing subacute and chronic non-malignant pain, highlights the urgent need for future research to develop effective nonopioid alternatives.

## Authors’ contributions

Study conceptualisation and pre-registration: SE, LAR, HBJ

Conceptualisation of the definitions of long- and short-term opioid use and resulting multistate model: all authors

Assistance with application for the data from the Norwegian Prescription Database: SOS

Data curation: OR, LAR, TN

Data access and verification: SE, MS, TN

Conceptualisation and completion of statistical analyses: SE, MS

Review of statistical analyses: TN, JMG

Securing funding and provision of necessary resources: LAR

Drafting the manuscript: SE

Critical review of the manuscript: all authors

Full access to all study data and taking responsibility for the decision to submit this manuscript for publication: all authors

## Data availability statement

As this study concerns data from Norwegian medical quality and health registries, data cannot be made publicly available. Written requests for data access or collaboration projects can be submitted to the head (LAR) of the Injury Prevention and Outcomes following Trauma (IPOT) project. Requests will be evaluated for relevance and eligibility by a committee of IPOT members. Additional approval from the Regional Committee for Medical and Health Research Ethics for working with deidentified IPOT data will, however, always be necessary. The study protocol, a simulated dataset, and the statistical code of the multistate model are publicly available at Open Science Framework (OSF) registries.^24^

## Declaration of generative AI and AI-assisted technologies in the writing process

During the preparation of this work, the authors used the Large Language Model ChatGPT 3.5-Turbo, hosted by the University of Oslo, to improve language and inspire statistical code. After using this tool, the authors reviewed and edited the content as needed and take full responsibility for the content of the publication.

## Funding

The present study constitutes part of the Injury Prevention and Outcomes following Trauma (IPOT) project, which has received funding from Oslo University Hospital (OUH), Norway. OUH did not have a role in design and conduct of the study, collection, management, analysis, and interpretation of the data, preparation, review, or approval of the manuscript, and decision to submit the manuscript for publication.

## Declarations of interest

HBJ is a co-owner of Bionics AS, a medical technology startup working on developing a vagus nerve stimulator for treating chronic pain. All other authors declare no conflicting interests.
