# Supplementary material for: Population-based multistate modelling of peritraumatic opioid use among trauma patients from the Norwegian Trauma Registry
Source: Br J Anaesth. 2025 Jul 4;135(2):390–400. doi: 10.1016/j.bja.2025.05.008 (PMC12308083; doi:10.1016/j.bja.2025.05.008)
Supplement: Multimedia component 1 [file mmc1.docx]

**Supplemental online content**

**Table of Contents**

[*Table S1*. Opioid types included in the present study and associated conversion ratios to calculate Morphine Milligram Equivalents (MME’s) 2](#_Toc194077625)

[*Figure S2*. Dispensation frequency and dispensed opioid types. 4](#_Toc194077626)

[*Figure S3*. Daily Morphine Milligram Equivalent (MME) supply for each quarter of the study period. 5](#_Toc194077627)

[*Table S4.* Detailed overview of patients’ daily Morphine Milligram Equivalent (MME) supply for each quarter of the study period 6](#_Toc194077628)

[*Figure S5*. Differences in Morphine Milligram Equivalents (MME) provided to patients with short- and long-term opioid use, categorized by injury severity. 7](#_Toc194077629)

[*Table S6.* Differences in the duration (days) of pre- and post-traumatic opioid use among long-term opioid use (LTU) patients based on injury severity 8](#_Toc194077630)

[*Table S7*. Sensitivity analysis: Comparisons of the 2015-2016 and 2017-2018 trauma patient cohorts on descriptive variables 9](#_Toc194077631)

[*Figure S8.* Sensitivity analysis: Multistatemodel of peritraumatic opioid use 11](#_Toc194077632)

[*Figure S9*. Sensitivity analysis: Multistatemodel of peritraumatic opioid use 12](#_Toc194077633)

[*Table S10*. Sensitivity analysis: Opioid types prescribed to adolescents (ages 12-17) 13](#_Toc194077634)

[*Table S11.* Sensitivity analysis: Comparison of peritraumatic opioid use among the complete sample (ages ≥12 years) and the adult subpopulation (ages ≥18 years) 14](#_Toc194077635)

# *Table S1*. Opioid types included in the present study and associated conversion ratios to calculate Morphine Milligram Equivalents (MME’s)

| **Opioids dispensed in Norway**  **2015-2020** | **ATC code** | **Form of Administration** | **DDD (mg)** | **Hamina et al.(2022)** ^1^ | **Norwegian Directorate of Health 2023/2024** ^2, 3^ | **Present publication** |
| --- | --- | --- | --- | --- | --- | --- |
| **Opioids used in opioid maintenance therapy** | | | | | | |
| Methadone | N07BC02 | PO | 25 | - | 6 | 6 |
| Levomethadone | N07BC05 | PO | 15 | - | - | 4 ^4, 5^ |
| Buprenorphine | N07BC01 | SL | 8 | - | 48 | 48 |
|  |  | Implant | 1.6 | - | - | - |
|  |  | P | 2.1 | - | 100 | 100 |
| Buprenorphine-naloxone | N07BC51 | SL | 8 | - | 48 | 48 |
| **Analgesic opioids** | | | | | | |
| Morphine | N02AA01 | PO | 100 | 1 | 1 | 1 |
|  |  | P | 30 | 3 | 3 | 3 |
|  |  | R | 30 | - | - | 1 ^6^ |
| Morphine combinations | N02AA51 | - | - | - | - | - |
| Hydromorphone | N02AA03 | PO | 20 | 3.6 | 5 | 5 |
|  |  | P | 4 | 15 | 15 | 15 |
|  |  | R | 4 | - | - | - |
| Oxycodone | N02AA05 | PO | 75 | 1.6 | 1.5 | 1.5 |
|  |  | P | 30 | 3 | 3 | 3 |
| Oxycodone and naloxone | N02AA55 | PO | 75 | 1.6 | - | 1.6 |
| Dihydrocodeine | N02AA08 | PO | 150 | - | 0.05 | 0.05 |
| Dihydrocodeine, combinations | N02AA58 | - | - | - | - | - |
| Ketobemidone | N02AB01 | PO | 50 | - | 1 | 1 |
|  |  | P | 50 | - | 3 | 3 |
| Ketobemidone  (incl. spasmolytic) | N02AG02 | PO | 5 | 2 | - | 2 |
|  |  | R | 10 | 0.3 | - | 0.3 |
| Pethidine | N02AB02 | PO | 400 | - | - | 0.1 ^7^ |
|  |  | R | 400 | 0.03 | 0.1 | 0.1 |
|  |  | P | 400 | 0.3 | 0.3 | 0.3 |
| Fentanyl | N02AB03 | SL | 0.6 | 200 | 250 | 250 |
|  |  | TD | 1.2 | 100 | 100 | 100 |
|  |  | P | 200 | 200 | 150 | 150 |
| Dextropropoxyphene (chloride) | N02AC04 | PO | 200 | 0.05 | - | 0.05 |
| Dextropropoxyphene (chloride) (combinations) | N02AC54 | PO | 70 | 0.05 | - | 0.05 |
| Pentazocine | N02AD01 | PO | 200 | 0.17 | - | 0.17 |
|  |  | P | 200 | 0.5 | - | 0.5 |
| Buprenorphine | N02AE01 | SL | 1.2 | 33 | 48 | 48 |
|  |  | P | 1.2 | 100 | - | 100 |
|  |  | TD | 1.2 | 110 | 92.5 | 110 |
| Codeine and paracetamol | N02AJ06 | PO | 90 | 0.05 | 0.1 | 0.1 |
|  |  | R | 90 | 0.05 | 0.1 | 0.1 |
| Codeine and acetylsalicylic acid | N02AJ07 | PO | 30 | 0.05 | - | 0.05 |
| Tramadol | N02AX02 | PO | 300 | 0.1 | 0.15 | 0.15 |
|  |  | R | 300 | - | - | 0.15* |
|  |  | P | 300 | 0.3 | - | 0.3 |
| Tramadol and paracetamol | N02AJ13 | P | 300 | 0.1 | - | 0.1 |
| Tapentadol | N02AX06 | PO | 400 | 0.1 | 0.2 | 0.2 |
| This table lists all opioids dispensed in Norway between 2014 and 2020 and included in the present study. For the opioids marked in grey associated data were neither found, nor were these dispensed during the study period.  DDD's were taken from https://www.whocc.no/atc_ddd_index/ and Hamina et al. (2022).^1^  **Abbreviations:** ATC, Anatomical Therapeutic Chemical; DDD, Defined Daily Dose; mg, milligrams; NTR, Norwegian Trauma Registry; PO, peroral; R, rectal; P, parenteral; SL, sublingual; TD, transdermal.  **Definition - Defined Daily Dose (DDD):** DDDs are a measurement unit reflecting the assumed average daily maintenance dose for adults using any respective ATC-classified drug for its main indication.^8^  **MME’s per dispensation** were calculated by multiplying the opioid-specific DDD with the associated conversion ratio and the number of DDD’s dispensed as registered in the Norwegian Prescription Database. | | | | | | |

**
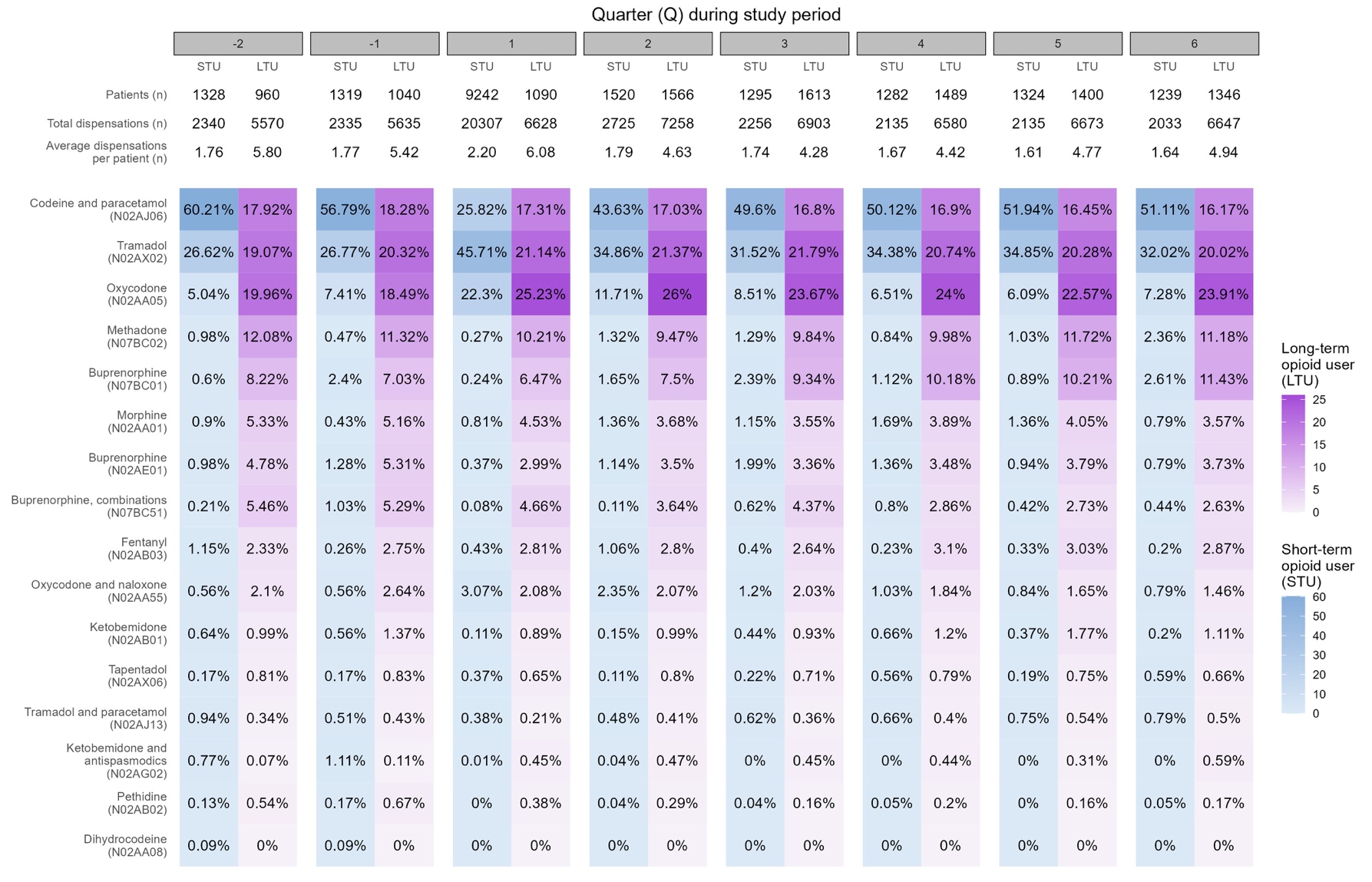
**

*Figure S2*. Dispensation frequency and dispensed opioid types. Opioid types supplied to study participants during the 180 days before and 620 days after injury are presented on the left side of the figure, sorted from most to least frequent. Per study quarter (90-day period), and opioid user type (short- and long-term opioid users), the average number of dispensations per individual and proportions of dispensations of listed opioid types are displayed.

***
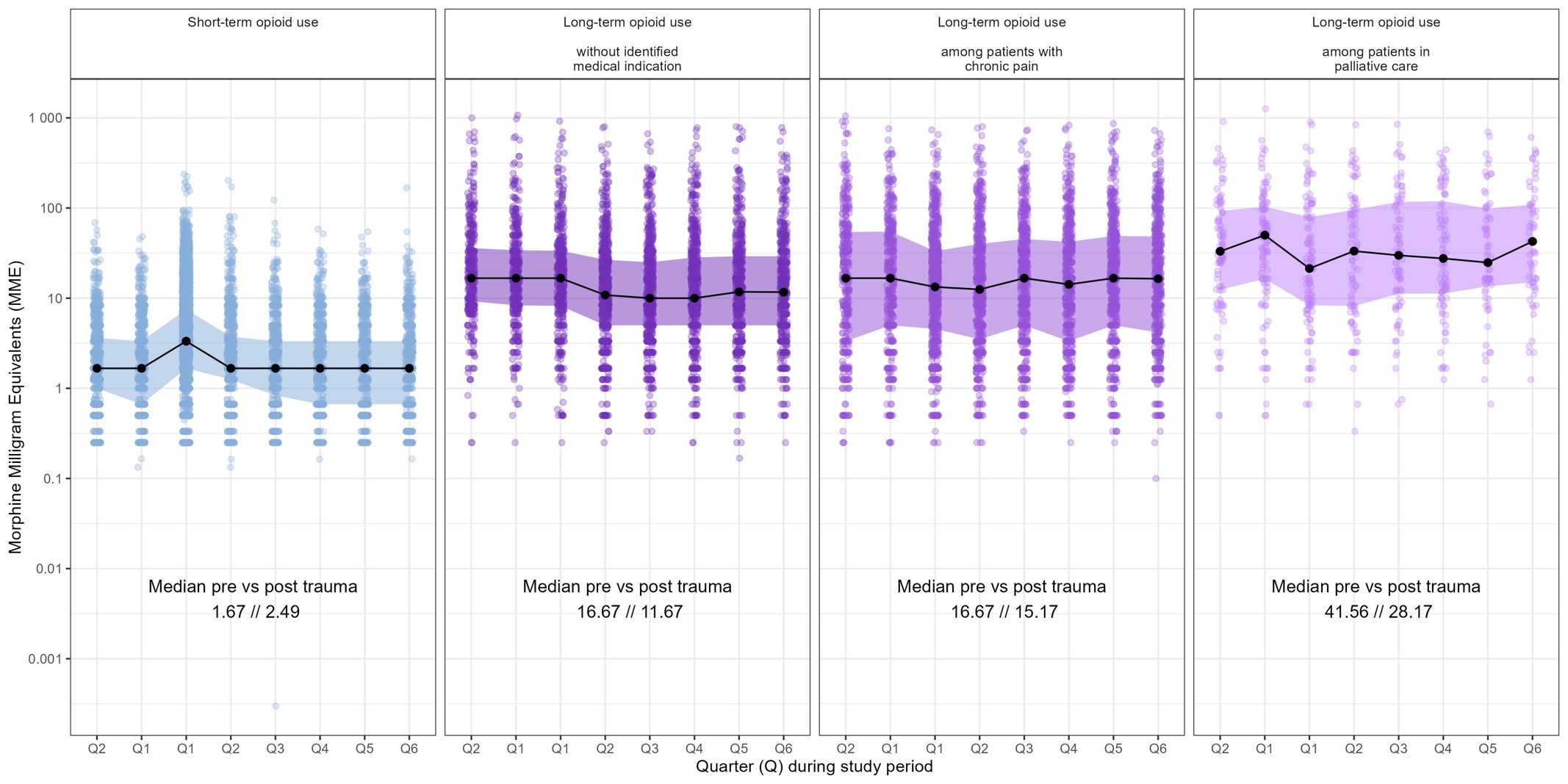
***

*Figure S3*. Daily Morphine Milligram Equivalent (MME) supply for each quarter of the study period. Individual patients’ daily MME supply for each of analyzed consecutive 90-day periods (Q) is displayed in jittered scatterplots. Black dots represent medians per quarter and shaded areas the interquartile range.

*Table S4.* Detailed overview of patients’ daily Morphine Milligram Equivalent (MME) supply for each quarter of the study period

|  | | | **Short-term opioid users** | | **Long-term opioid users** | | | | | |
| --- | --- | --- | --- | --- | --- | --- | --- | --- | --- | --- |
|  |  |  |  |  | **Without identified medical indication** | | **Patients with chronic pain** | | **Patients in palliative care** | |
|  |  |  | M ± SD | Median (25^th^, 75^th^) | M ± SD | Median (25^th^, 75^th^) | M ± SD | Median (25^th^, 75^th^) | M ± SD | Median (25^th^, 75^th^) |
| Time from trauma (Quarter) | Pre | 2 | 3.37 ± 5.20 | 1.67 (1.00, 3.62) | 44.30 ± 94.29 | 16.67 (9.25, 35.94) | 58.91 ± 127.47 | 16.67 (3.33, 54.19) | 81.48 ± 129.96 | 33.09 (12.41, 92.46) |
|  |  | 1 | 3.03 ± 4.32 | 1.67 (0.67, 3.33) | 41.83 ± 95.11 | 16.67 (8.33, 34.00) | 48.02 ± 84.61 | 16.67 (500, 54.93) | 91.21 ± 153.01 | 50.00 (16.43, 103.42) |
|  | Post | 1 | 6.34 ± 10.38 | 3.33 (1.67, 7.48) | 39.31 ± 83.73 | 16.67 (8.21, 33.61) | 32.87 ± 63.34 | 13.33 (4.56, 33.21) | 73.88 ± 140.85 | 21.33 (8.33, 79.28) |
|  |  | 2 | 4.43 ± 10.81 | 1.67 (1.25, 3.75) | 28.55 ± 63.53 | 10.83 (5.00, 26.67) | 40.37 ± 80.95 | 12.50 (3.50, 40.00) | 84.09 ± 129.41 | 33.33 (8.17, 95.59) |
|  |  | 3 | 3.30 ± 6.28 | 1.67 (0.83, 3.33) | 28.24 ± 58.52 | 10.00 (5.00, 25.00) | 42.18 ± 76.95 | 16.67 (5.00, 45.29) | 88.28 ± 140.30 | 29.83 (11.22, 116.90) |
|  |  | 4 | 3.05 ± 4.55 | 1.67 (0.67, 3.33) | 33.40 ± 73.56 | 10.00 (5.00, 28.33) | 42.26 ± 84.85 | 14.22 (3.33, 42.23) | 82.12 ± 106.34 | 27.75 (11.25, 119.02) |
|  |  | 5 | 3.01 ± 4.23 | 1.67 (0.67, 3.33) | 33.96 ± 81.70 | 11.75 (5.00, 29.17) | 46.76 ± 94.39 | 16.67 (5.00, 48.99) | 86.11 ± 138.15 | 24.82 (13.58, 98.50) |
|  |  | 6 | 3.17 ± 6.77 | 1.67 (0.67, 3.33) | 33.96 ± 73.44 | 11.67 (5.00, 29.17) | 44.67 ± 81.58 | 16.43 (4.17, 48.33) | 97.72 ± 132.11 | 42.58 (15.00, 109.07) |
| This table provides the exact numbers underlying Figure S3.  **Abbreviations:** M, mean; SD, standard deviation; 25^th^, 25^th^ percentile; 75^th^, 75^th^ percentile | | | | | | | | | | |

***
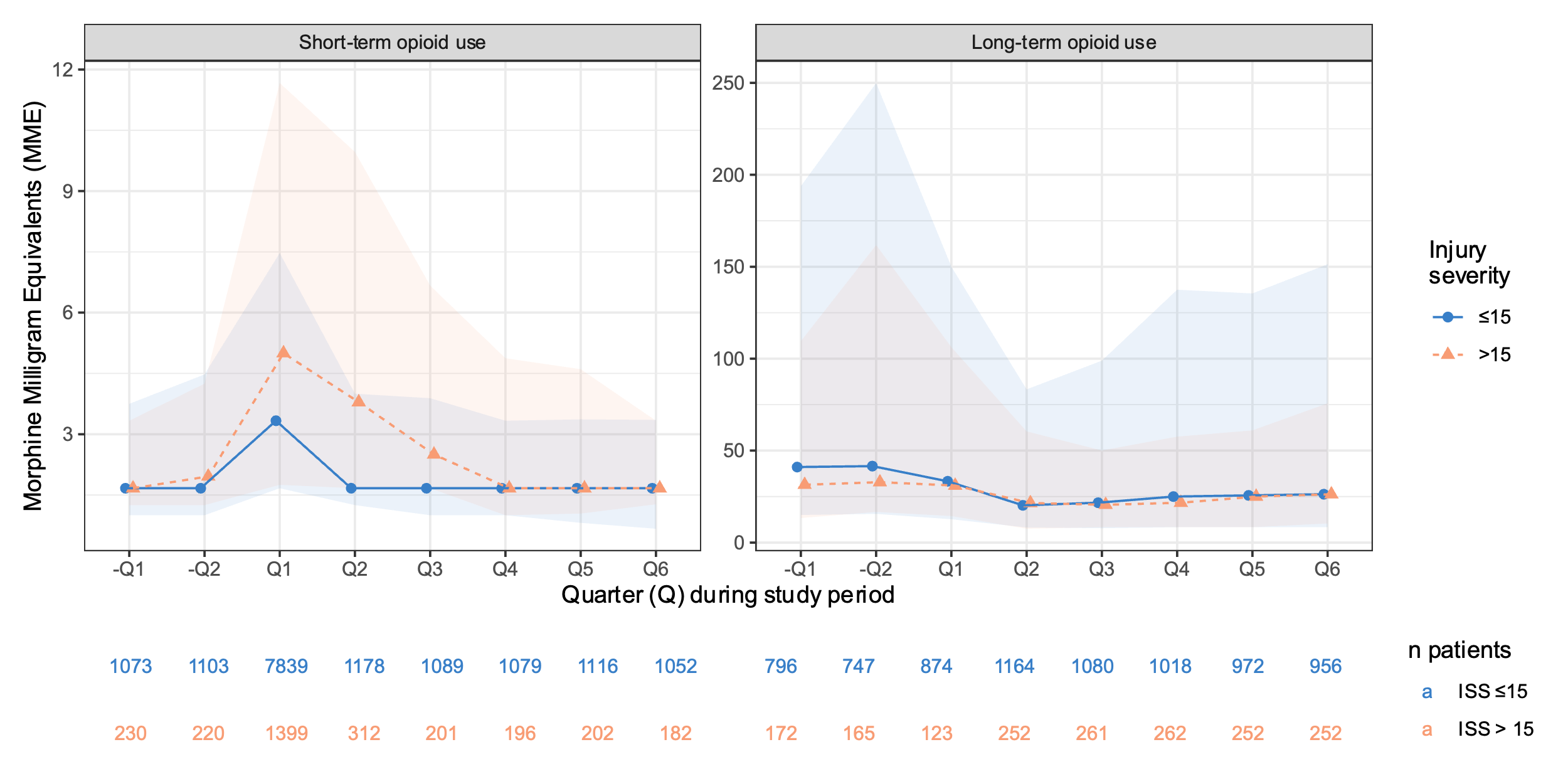
***

# *Figure S5*. Differences in Morphine Milligram Equivalents (MME) provided to patients with short- and long-term opioid use, categorized by injury severity.

This figure presents the median MME per study quarter (90 days) for patients with short- and long-term opioid use, classified by injury severity: non-severe injuries (Injury Severity Score (ISS) ≤15, shown in blue) and severe injuries (ISS >15, shown in orange). The shaded areas depict the interquartile ranges for each respective group. Below the line graphs representing short-term and long-term opioid users, the number of patients (n) with non-severe and severe injuries is provided for each study quarter.

# *Table S6.* Differences in the duration (days) of pre- and post-traumatic opioid use among long-term opioid use (LTU) patients based on injury severity

| **Injury severity** | **Before trauma (days)** | | **After trauma (days)** | |
| --- | --- | --- | --- | --- |
|  | M (SD) | Median (25^th^, 75^th^) | M (SD) | Median (25^th^, 75^th^) |
| **Non-severe (ISS ≤15)** | 152 (49.6) | 180 (146, 180) | 332 (185) | 358 (145, 540) |
| **Severe (ISS >15)** | 156 (47.1) | 180 (158, 180) | 267 (173) | 248 (94.2, 398) |
| **Abbreviations:** M, mean; SD, standard deviation; 25^th^, 25^th^ percentile; 75^th^, 75^th^ percentile; ISS, Injury Severity Score | | | | |

# *Table S7*. Sensitivity analysis: Comparisons of the 2015-2016 and 2017-2018 trauma patient cohorts on descriptive variables

| **Variable** | **NTR Cohort** | | | | **Abs Δ** | **Statistical test**  **t(*df*)/ χ^2^(*df, N*)** | ***P*** | ***d’ or V*** |
| --- | --- | --- | --- | --- | --- | --- | --- | --- |
|  | **2015-2016** (n=11,621) | | **2017-2018** (n=12,601) | |  |  |  |  |
|  | M(SD)/N(%) | Median(25^th^, 75^th^) | M (SD)/N(%) | Median(25^th^, 75^th^) |  |  |  |  |
| **Demographics** | | | | | | | | |
| Age (years) | 45.11 (21.92) | 44 (25, 62) | 45.96 (22.35) | 45 (25, 63) | 0.85 | t(24128,745) = -2.961 | .003 | 0.038 |
| Gender | | | | | | | | |
| Female | 3829 (33.0%) | NA | 3977 (31.6%) | NA | 1.4 | $\chi^{2}\left( 1,24224 \right)=5.195$ | .023 | 0.015 |
| Male | 7793 (67.1%) | NA | 8625 (68.4%) | NA | 1.3 |  |  |  |
| **Somatic comorbidity** | | | | | | | | |
| ASA 1 | 7213 (62.1%) | NA | 7427 (59.0%) | NA | 3.1 | $\chi^{2}\left( 2, 23612 \right)=$ 30.328 | <.001 | 0.036 |
| ASA 2 | 2904 (25.0%) | NA | 3523 (28.0%) | NA | 3.0 |  |  |  |
| ASA ≥3 | 1205 (10.4%) | NA | 1340 (10.6%) | NA | 0.2 |  |  |  |
| *Missing* | 300 (2.6%) | NA | 312 (2.5%) | NA | 0.1 |  |  |  |
| **Mechanism of injury** | | | | | | | | |
| Traffic-related | 5527 (47.6%) | NA | 5825 (46.2%) | NA | 1.4 | $\chi^{2}\left( 2, 23569 \right)=8.709$ | .013 | 0.019 |
| Fall | 3959 (34.1%) | NA | 4534 (36.0%) | NA | 1.9 |  |  |  |
| Other | 1800 (15.5%) | NA | 1926 (15.3%) | NA | 0.2 |  |  |  |
| *Missing* | 336 (2.9%) | NA | 317 (2.5%) | NA | 0.4 |  |  |  |
| **Injury characteristics** | | | | | | | | |
| ISS | 8.33 (9.00) | 5 (1, 10) | 10.76 (11.90) | 6 (2, 16) | 0.30 | t(23827.322) = 2.739 | .006 | 0.035 |
| NISS | 8.03 (8.60) | 5 (1, 10) | 10.40 (11.51) | 6 (2, 14) | 0.37 | t(23900.745) = 2.471 | .013 | 0.035 |
| Injured body part according to AIS (AIS ≥3) | | | | | | | | |
| Head | 1578 (13.6%) | NA | 1536 (12.2%) | NA | 1.4 | $\chi^{2}\left( 1, 24224 \right)=10.291$ | .001 | 0.021 |
| Face | 68 (0.6%) | NA | 60 (0.5%) | NA | 0.1 | $\chi^{2}\left( 1, 24224 \right)=$ 1.167 | .280 | 0.007 |
| Neck | 54 (0.5%) | NA | 52 (0.4%) | NA | 0.1 | $\chi^{2}\left( 1, 24224 \right)=$ 0.265 | .606 | 0.003 |
| Thorax | 1541 (13.3%) | NA | 1670 (13.2%) | NA | 0.1 | $\chi^{2}\left( 1, 24224 \right)=$ 0.000 | 1.000 | 0.000 |
| Abdomen | 412 (3.6%) | NA | 409 (3.3%) | NA | 0.3 | $\chi^{2}\left( 1, 24224 \right)=1.566$ | .211 | 0.008 |
| Spine | 467 (4.0%) | NA | 499 (4.0%) | NA | 0.0 | $\chi^{2}\left( 1, 24224 \right)=$ 0.040 | .842 | 0.001 |
| Upper extremities | 104 (0.9%) | NA | 112 (0.9%) | NA | 0.0 | $\chi^{2}\left( 1, 24224 \right)=0.000$ | 1.000 | 0.000 |
| Lower extremities | 795 (6.8%) | NA | 860 (6.8%) | NA | 0.0 | $\chi^{2}\left( 1, 24224 \right)=$ 0.000 | 1.000 | 0.000 |
| **Hospital stay** | | | | | | | | |
| ICU admission | 6766 (58.2%) | NA | 7162 (56.8%) | NA | 1.4 | $\chi^{2}\left( 1, 23790 \right)=4.041$ | .044 | 0.013 |
| *Missing* | 198 (1.7%) | NA | 235 (1.9%) | NA | 0.2 |  |  |  |
| Length ICU stay (days) | 1.68 (3.90) | 1 (0, 2) | 1.56 (3.80) | 1 (0, 2) | 0.12 | t(23529.320) = 2.522 | .012 | 0.033 |
| *Missing* | 198 (1.7%) | NA | 235 (1.9%) |  | 0.2 |  |  |  |
| Surgery within 2 weeks from trauma | 4156 (35.8%) | NA | 4346 (34.5%) | NA | 1.3 | $\chi^{2}\left( 1, 24224 \right)=4.248$ | .039 | 0.013 |
| Length hospital stay (days) | 2.80 (6.92) | 1 (0, 3) | 2.94 (6.37) | 2 (0, 3) | 0.12 | t(23331.956) = -1.661 | 0.097 | 0.022 |
| *Missing* | 116 (1.0%) | NA | 160 (1.3%) | NA | 0.3 |  |  |  |
| **Abbreviations:** M, mean; SD, standard deviation; N, number of patients; 25^th^, 25^th^ percentile; 75^th^, 75^th^ percentile; Abs Δ, absolute difference; t, t-value; $\chi^{2}$, chi-square statistic; *df,* degrees of freedom; p, p-value; *d,* Cohen’s d; V, Cramer’s V; ASA, American Society of Anesthesiologists physical status classification system; ISS, Injury Severity Score; NISS, New Injury Severity Score; AIS, Abbreviated Injury Scale; ICU, Intensive Care Unit  Where applicable, numbers of missing cases are indicated.  **Interpretation Cohen’s d:** 0.2 = small effect, 0.5 = medium effect, 0.8 = large effect ^9^ **Interpretation Cramer’s V one degree of freedom:** 0.1 = weak effect, 0.3 = moderate effect, 0.5 = strong effect ^10^ **Interpretation Cramer’s V two degrees of freedom:** 0.07 = weak effect, 0.21 = moderate effect, 0.35 = strong effect ^10^ | | | | | | | | |

**
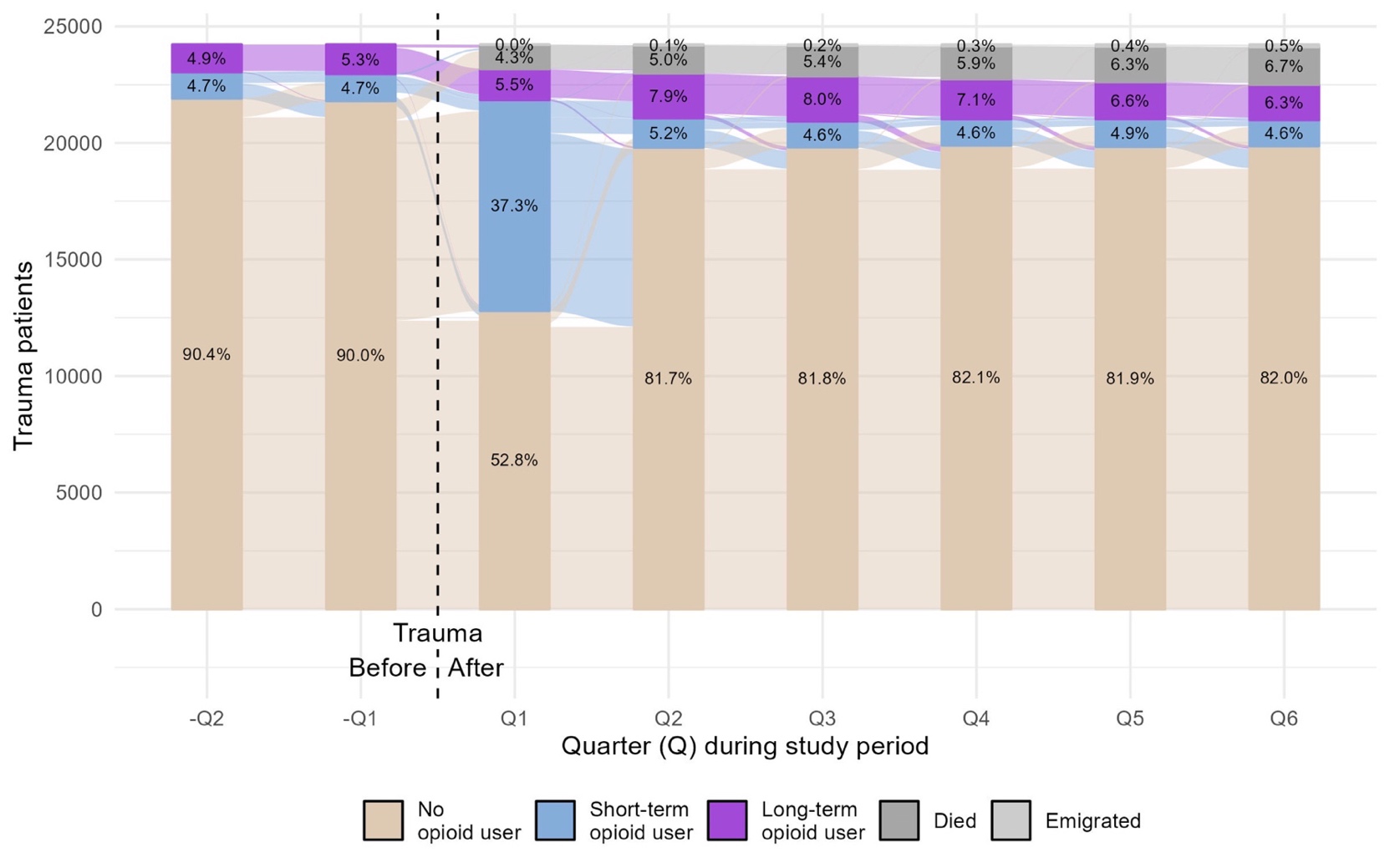
**

*Figure S8.* Sensitivity analysis: Multistatemodel of peritraumatic opioid use **among 24,224 Norwegian trauma patients** using a criterion of ≥450 Morphine Milligram Equivalents (MME) for the initial 90 days of long-term opioid use. This Alluvial plot depicts how the progression of opioid use patterns (no, short-term, long-term) and competing events (death, emigration) unfolds from 6 months prior to 18 months post-trauma when the MME criterion for long-term opioid use in the first 90 days is reduced from ≥10 MME to ≥5 MME per day (hence from ≥900 MME to ≥450 MME for the first 90 days). The X-axis represents time in quarters (90-day periods), while the Y-axis shows the total number of patients. Each bar indicates the prevalence of different opioid use patterns and competing events per quarter. The flows between bars depict transitions of patients between states from one quarter to the next. Each flow is color-coded based on the originating category and directed toward the destination category. The width of each flow corresponds to the number of patients making that specific transition; wider flows represent more frequent category changes.

**
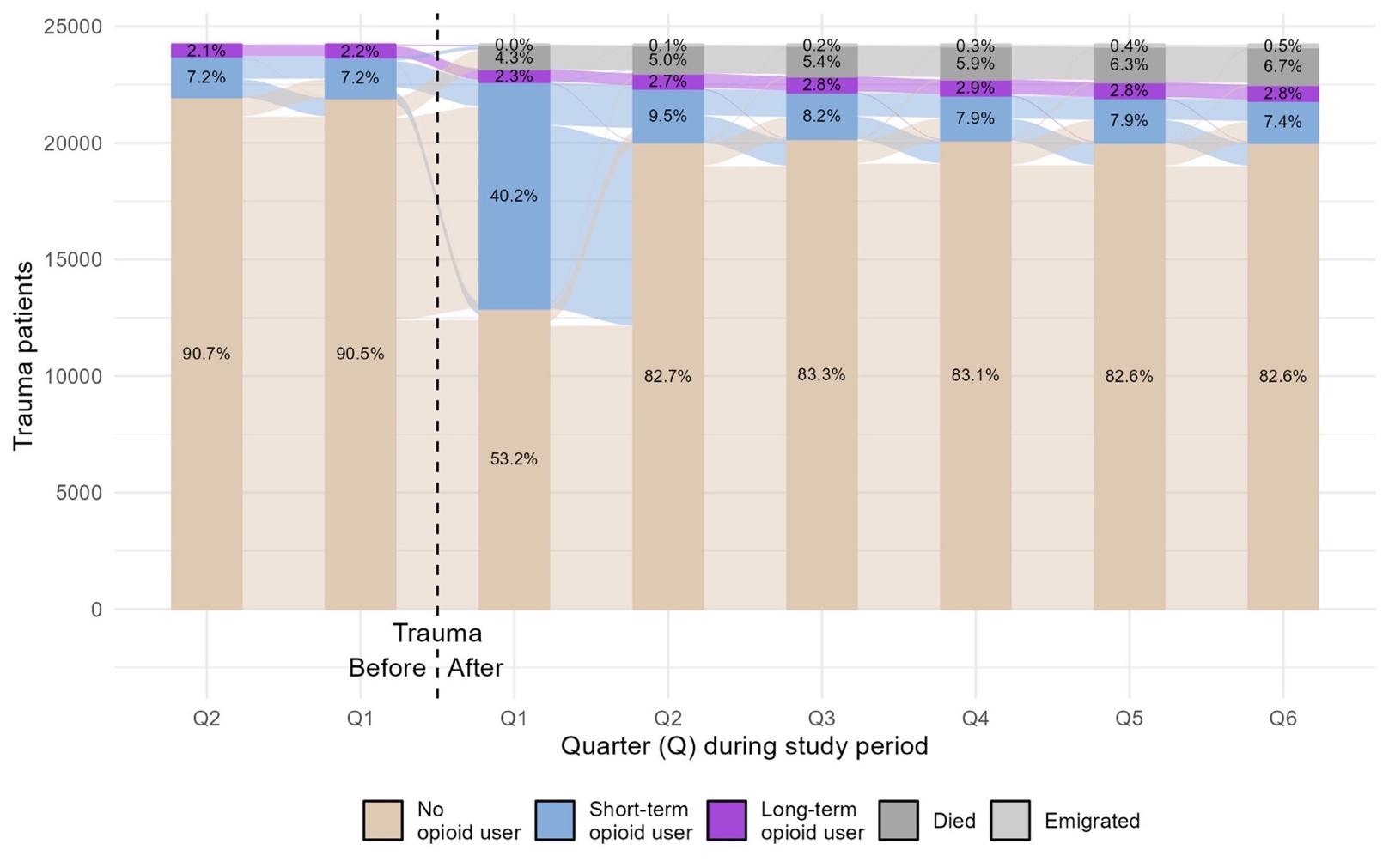
**

*Figure S9*. Sensitivity analysis: Multistatemodel of peritraumatic opioid use **among 24,224 Norwegian trauma patients** using a criterion of ≥4500 Morphine Milligram Equivalents (MME) for the initial 90 days of long-term opioid use. This Alluvial plot depicts how the progression of opioid use patterns (no, short-term, long-term) and competing events (death, emigration) unfolds from 6 months prior to 18 months post-trauma when the MME criterion for long-term opioid use in the first 90 days is increased from ≥10 MME to ≥50 MME per day (hence from ≥900 MME to ≥4500 MME for the first 90 days). The X-axis represents time in quarters (90-day periods), while the Y-axis shows the total number of patients. Each bar indicates the prevalence of different opioid use patterns and competing events per quarter. The flows between bars depict transitions of patients between states from one quarter to the next. Each flow is color-coded based on the originating category and directed toward the destination category. The width of each flow corresponds to the number of patients making that specific transition; wider flows represent more frequent category changes.

# *Table S10*. Sensitivity analysis: Opioid types prescribed to adolescents (ages 12-17)

| **Opioid type** | **ATC code** | **Administration method** | **Standard dose according to Norwegian prescription guidelines**^11, 12^ | **Proportion of dispensations to adolescents** | |
| --- | --- | --- | --- | --- | --- |
|  |  |  |  | **Pre-trauma** | **Post-trauma** |
| **Morphine** | N02AA01 | PO | 0.4-1.6 mg/kg/day divided into 2 doses. Starting dose in opioid-naive patients is max. 60 mg/day. | 0% | 0.25% |
| **Oxycodone, OxyNorm** | N02AA05 | PO | 20-30 mg/day divided into 4 - 6 doses. | 7.5% | 11.47% |
| **Oxycodone, OxyContin** |  | PO | Starting dose in opioid-naive patients: 20 mg/day divided into 2 doses. |  |  |
| **Tramadol** | N02AX02 | PO | 50-100 mg/dose as needed, up to 4 times daily. Maximum: 400 mg/day. | 20.0% | 48.12% |
| **Tapentadol** | N02AX06 | PO | Starting dose in opioid-naive patients: 1.5 mg/kg body weight every 12 hours. Should not exceed 50 mg. | 5.0% | 2.1% |
| **Codeine** | N02AJ06 | PO | 30 mg codeine per dose, up to a maximum of 4 times daily. Dosing interval 6 hours. | 67.5% | 38% |
| **Abbreviations:** ATC, Anatomical Therapeutic Chemical; PO, per os (orally)  Since none of the adolescents aged 12 to 17 received parenteral opioids, it was decided to include only oral dosage information in this table.  For comparison: A 12-year-old adolescent at the 5th percentile weighs 30-31kg, while one at the 50th percentile weighs 40-41 kg.^13^ | | | | | |

# *Table S11.* Sensitivity analysis: Comparison of peritraumatic opioid use among the complete sample (ages ≥12 years) and the adult subpopulation (ages ≥18 years)

|  | | | **Complete sample (n= 24,224)** | | | **Adults only (≥18 years; n= 22,037 )** | | | **Cramer’s V** |
| --- | --- | --- | --- | --- | --- | --- | --- | --- | --- |
|  |  |  | **NU** | **STU** | **LTU** | **NU** | **STU** | **LTU** |  |
| Time from trauma (Quarter) | Pre | 2 | 90.6% | 5.5% | 4.0% | 89.7% | 6.0% | 4.4% | 0.010 |
|  |  | 1 | 90.3% | 5.4% | 4.3% | 89.4% | 5.9% | 4.7% | 0.010 |
|  | Post | 1 | 53.0% | 38.2% | 4.5% | 50.1% | 40.3% | 4.9% | 0.014 |
|  |  | 2 | 82.1% | 6.3% | 6.5% | 80.6% | 6.7% | 7.1% | 0.009 |
|  |  | 3 | 82.4% | 5.3% | 6.7% | 80.8% | 5.8% | 7.3% | 0.010 |
|  |  | 4 | 82.4% | 5.3% | 6.1% | 80.9% | 5.7% | 6.7% | 0.009 |
|  |  | 5 | 82.1% | 5.5% | 5.8% | 80.6% | 5.8% | 6.3% | 0.009 |
|  |  | 6 | 82.2% | 5.1% | 5.6% | 80.7% | 5.5% | 5.1% | 0.009 |
| **Abbreviations:** NU, no opioid use; STU, short-term opioid use; LTU, long-term opioid use.  Cramer's V was calculated to quantify the magnitude of the differences between the two samples.  **Interpretation Cramer’s V -Q1 and -Q2, two degrees of freedom:** 0.07 = weak effect, 0.21 = moderate effect, 0.35 = strong effect^10^  **Interpretation Cramer’s V Q1 to Q6, four degrees of freedom: :** 0.05 = weak effect, 0.15 = moderate effect, 0.25 = strong effect^10^  Since the proportions of patients who emigrated or died are not shown, the percentages for the posttraumatic quarters do not total 100%. | | | | | | | | | |

**References**

1. Hamina A, Hjellvik V, Handal M, Odsbu I, Clausen T, Skurtveit S. Describing long-term opioid use utilizing Nordic prescription registers—A Norwegian example. *Basic & Clinical Pharmacology & Toxicology* 2022; **130**: 481–91

2. The Norwegian Directorate of Health. Ekvipotenser for opioider [Equipotencies for opioids] [Internet]. Helsedirektoratet. [cited 2024 Oct 3]. Available from: https://www.helsedirektoratet.no/veiledere/vanedannende-legemidler/pasientinformasjon-og-verktoy/konvertering-av-opioiddoser

3. The Norwegian Directorate of Health. Omregningstabell for opioider til orale morfinekvivalenter (OMEQ) [Conversion table for opioids to oral morphine equivalents (OMEQ)] [Internet]. Helfo - for helseaktører. [cited 2024 Aug 18]. Available from: https://www.helfo.no/lege/blaareseptordningen/omregningstabell-for-opioider-til-orale-morfinekvivalenter-(OMEQ)

4. Steins MB, Eschbach C, Villalobos M, Thomas M. Schmerztherapie in der Palliativmedizin [Pain Management in Palliative Care]. 2018; Available from: https://www.thieme-connect.com/products/ejournals/pdf/10.1055/s-0043-122667.pdf

5. Drug Commission of the German Medical Association. Schmerztherapie bei Tumorpatienten in der (fortgeschrittenen) Palliativsituation [Pain therapy for tumor patients in the (advanced) palliative situation]. 2020; Available from: https://www.akdae.de/fileadmin/user_upload/akdae/Arzneimitteltherapie/AVP/vorab/20200803-Tumorschmerztherapie.pdf

6. Vold JH, Skurtveit S, Aas C, Johansson KA, Fadnes LT. Too much or too little opioids to patients receiving opioid agonist therapy in Norway (2013–2017): a prospective cohort study. *BMC Health Serv Res* 2020; **20**: 668

7. Svendsen K, Borchgrevink P, Fredheim O, Hamunen K, Mellbye A, Dale O. Choosing the unit of measurement counts: the use of oral morphine equivalents in studies of opioid consumption is a useful addition to defined daily doses. *Palliat Med* 2011; **25**: 725–32

8. World Health Organization. WHO - Definition and general considerations - Basic definition of the defined daily dose (DDD). [Internet]. [cited 2024 Aug 18]. Available from: https://atcddd.fhi.no/ddd/definition_and_general_considera/

9. Lakens D. Calculating and reporting effect sizes to facilitate cumulative science: a practical primer for t-tests and ANOVAs. *Front Psychol* [Internet] Frontiers; 2013 [cited 2024 Aug 18]; **4** Available from: https://www.frontiersin.org/journals/psychology/articles/10.3389/fpsyg.2013.00863/full

10. Kim H-Y. Statistical notes for clinical researchers: Chi-squared test and Fisher’s exact test. *Restor Dent Endod* 2017; **42**: 152–5

11. Koble [Internet]. [cited 2025 Mar 8]. Available from: https://koble.info/

12. Medisin - Felleskatalogen [Internet]. [cited 2025 Mar 8]. Available from: https://www.felleskatalogen.no/medisin

13. Growth Charts - Clinical Growth Charts [Internet]. 2024 [cited 2025 Mar 8]. Available from: https://www.cdc.gov/growthcharts/cdc-charts.htm
